# Editorial: Immunotherapy in renal cell carcinoma

**DOI:** 10.3389/fonc.2022.945510

**Published:** 2022-07-27

**Authors:** Viktor Gruenwald, Walter J. Storkus

**Affiliations:** ^1^ Interdisciplinary Genitourinary Oncology, Clinic for Medical Oncology and Clinic for Urology, University Hospital Essen, Essen, Germany; ^2^ Dermatology, Immunology, Pathology and Bioengineering, University of Pittsburgh School of Medicine and the University of Pittsburgh Medical Center (UPMC) Hillman Cancer Center, Pittsburgh, PA, United States

**Keywords:** immunotherapy, cancer, renal cell carcinoma (RCC), biomarker, urologic oncology

## Abstract

This collection contains 10 reports published In Frontiers in Oncology between August 2020 and March 2022 broadly focused on the immunobiology of renal cell carcinoma (RCC), the impact of immunotherapy in the setting of RCC, and the identification of biomarkers that are prognostic of RCC patient outcomes and response to immunotherapy.

## Introduction

Renal cell carcinoma (RCC) accounts for 90-95% of all kidney malignancies. If detected early and managed surgically, the 5-year overall survival rate for RCC patients is ~90% (1). However, one-third of treated patients develop disease recurrence or metastases. Patients diagnosed with advanced-stage metastatic disease have a very poor prognosis, with a 5-year survival rate of 14% in a real-world population (2). There remains a clear and unmet clinical need for the development of effective interventional therapies for RCC patients, particularly for those individuals with advanced-stage disease.

Cytokine-based immunotherapies were developed as standard-of-care treatments for metastatic RCC patients in the 1990s. However, only a minority of patients derived durable clinical benefits. With the advent of refined molecular profiling of RCC, the field witnessed the rapid evolution of targeted therapeutic approaches yielding improved response rates in the early 2000s, with more recent advances (since 2013) including the application of immune checkpoint inhibitors (ICI) to enhance and sustain anti-tumor immune cell function in association with extended progression-free and overall survival in treated RCC patients (1, 3). More recently, ICI-combinations have gained momentum and represent a 1^st^ line standard of care (4), although much work remains to improve current rates of response to ICI-based treatments while coordinately limiting immune-related adverse events (irAEs). In this regard, biomarkers (at baseline and on-treatment) associated with patient response to immunotherapy are also expected to improve patient diagnoses and therapeutic management, while coordinately serving as monitoring tools to assess patient response to treatment in real-time (5–7).

The current collection advances our understanding of clinically informative biomarkers relevant to patient care and the safety/impact of interventional immunotherapies in the setting of urologic oncology.

## RCC immune-associated prognostic biomarkers

Du et al. analyzed immune cell-associated transcripts in the clear cell renal cell carcinoma (ccRCC; KIRK) TCGA database, identifying 7 immune cell type gene signatures associated with patient overall survival (OS). Abundant B (memory, plasma) cells, Treg and M1 macrophage transcripts were linked to poor prognosis, while elevated levels of dendritic cell (DC; resting, activated) and resting mast cells were correlated to extended OS. These data are consistent with higher abundance ratios of DC and activated DC in tumors of patients with better OS and presumed enhanced antigen-presenting cell performance. Low or high expression of 7 HUB genes in the TME also appeared prognostic of prolonged patient OS: BDKRB1 (low), CASR (high), GNG4 (low), MFI2 (low), MMP9 (low), NUM (low) and SAA1 (low). Additional immune-related genes were enriched in neuroactive ligand-receptor binding, cytokine/cytokine receptor interactions, GPCR ligand binding, and APC function that may represent useful prognostic indices and serve to define novel targets for developing prospective interventional approaches.

Long non-coding RNAs (lncRNA) are long RNAs (> 200 nucleotides) that play roles in chromatin remodeling, as well as transcriptional and post-transcriptional gene regulation (Sun et al.). In this collection, (Sun et al.) developed a novel immune-related lncRNA signature based on an analysis of the TCGA KIRC database for prognosis of ccRCC patients. They identified 5 prognostic lncRNAs (3 harmful, 2 protective), which appeared to serve as independent predictors of tumor stage and high-risk (short OS) vs. low-risk (long OS) patient status. Four of these lncRNAs were upregulated in the TME with predicted impact on local expression of immune checkpoint molecules (PD-1, PD-L1, CTLA4), immunomodulatory cytokines, as well as TCR, Wnt/β-catenin and MAPK signaling.

A meta-analysis performed by (Jin et al.) evaluated the systemic immune index (SII) based on neutrophil, platelet, and lymphocyte counts in peripheral blood PBL as a prognostic index in ccRCC patients. High SII was independently associated with aggressive disease and poor OS, but not PFS or cancer-specific survival.

Ferroptosis is a form of immunogenic cell death believed to support improved immune cell recognition of tumor cells (Gao et al.). In this regard, (Gao et al.) described a novel ferroptosis-related 4-gene signature (BID, MT1G, SLC7A11, TAZ) that appears diagnostic/prognostic in ccRCC patients. Notably, TAZ also appears to modulate regulatory factors in the TME favoring recruitment/development of suppressor cells (TAMs, Treg) and upregulated expression of immune checkpoint molecules. High- (poor OS) and low-risk groups based on this 4 gene signature (FeSig) could be distinguished based on differences in immune pathways (Ras-, PPAR- and IL-17A-signaling) and immune cell (macrophages, mast cells, PMNs, CD4^+^ T cells [Th1, Th2, Tfh], and CD8^+^ T cells) content within the TME. Expression of PD-1, CTLA4, LAG3, and TIGIT was upregulated in high-risk vs. low-risk patients, with high-risk patients exhibiting superior response to anti-PD-1-based monotherapy. Based on these findings, future immunotherapies coordinately targeting ICI and the TAZ/WNT10B signaling axis are proposed for improved outcomes in ccRCC patients.

Nie et al. report that transcripts for Fcγ receptors (FCGR; FCGR1A/B/C, FCGR2A) were upregulated in ccRCC tumors based on differential gene methylation, where they were linked to tumor grade/stage and associated with poor patient OS. CD4^+^ T and NK were negatively-, while Treg and M2 macrophage cells were positively-, correlated with FCGR transcript expression levels. Additionally, ccRCC tumors with high FCGR expression exhibited increased expression of immune suppressor/regulatory molecules IL-10, TGFB1 and CTLA4. These results suggest that high tumor expression of FCGRs defines a risk factor for ccRCC patient survival associated with poor prognosis and regulatory immune status in the TME.

## RCC immunobiology and immunotherapy

Kuo et al. retrospectively evaluated the safety and efficacy of ICIs (anti-PD-1, anti-PD-L1, anti-CTLA4) in patients with metastatic urothelial carcinoma with/without end-stage renal disease (ESRD). Although small in sample size, the study reports a higher overall response rate (ORR) for ESRD vs. non-ESRD patients (55% vs. 29%), with 6 PR/1 SD documented amongst the 11 ESRD patients treated. Potential prognostic factors in multivariate analyses included leukocytosis and neutrophil-to-lymphocyte ratio.

Stellato et al. retrospectively evaluated the impact of prior nephrectomy on response to ICI-based intervention in 287 metastatic RCC patients. Multivariate analyses revealed that prior nephrectomy was associated with superior patient OS and PFS. These data are consistent with superior outcomes for advanced-stage mRCC patients treated with biologic modifiers IL-2/IFNA (or anti-PD1/CTLA4 vs. sunitinib in the Checkmate214 trial).

Gopalakrishanan et al. provide a case report in which anti-PD1 triggered (over)exuberant tumor inflammation in association with radiologic hyper-progression and clinical deterioration in 3 patients with advanced ccRCC with diverse visceral sites of disease (Gopalakrishnan et al.). Histologic examination of tumors revealed tissue necrosis with lymphohistiocytic infiltration and evidence of robust immune-mediated tumor-associated killing and macrophage scavenging of debris. The authors suggest these conditions promote the rapid expansion of the anti-tumor T cell repertoire, pro-inflammatory immune cell infiltration and cytokine-release syndrome. To mitigate such immune cell over-reactivity to ICI-based treatment, the authors suggest use of combination regimens including IL-6i (tocilizumab).

Wang et al. evaluated the rare autosomal dominant disorder hereditary leiomyomatosis and RCC (HLRCC) in which patients are at risk of developing multiple skin and uterine leiomyomas and RCC with poor clinical outcomes based on deletion of the tumor suppressor gene fumarate hydratase (FH), an enzyme in the TCA cycle (Wang et al.). While this rare disease has no currently effective standard of care, the authors provide a case study for HLRCC patients who developed a complete response in their PD-L1^+^PD-1^+^ tumors after 24 months of treatment with anti-PD1 monotherapy.

Zhou et al. provide a case report and literature review for treatment with tyrosine kinase inhibitors (TKI; pazopanib, axitinib) and ICI in the setting of a collecting duct renal carcinoma (CDC), a rare (0.4-2.0% of RCC cases) and highly aggressive subtype of kidney cancer with poor prognosis with limited effective therapies. In this setting, combination immunotherapy with TKI + ICI after cytoreductive nephrectomy resulted in a clinical PR and extended OS in a patient with CDC, supporting more general testing of such interventional approaches in the future.

## Author contributions

All authors listed have made a substantial, direct, and intellectual contribution to the work and approved it for publication.

## Funding

This work was supported in part by NIH grant R01 CA249811 (to WJS).

## Conflict of interest

The authors declare that the research was conducted in the absence of any commercial or financial relationships that could be construed as a potential conflict of interest.

## Publisher’s Note

All claims expressed in this article are solely those of the authors and do not necessarily represent those of their affiliated organizations, or those of the publisher, the editors and the reviewers. Any product that may be evaluated in this article, or claim that may be made by its manufacturer, is not guaranteed or endorsed by the publisher.
